# An Acoustic Signal Enhancement Method Based on Independent Vector Analysis for Moving Target Classification in the Wild

**DOI:** 10.3390/s17102224

**Published:** 2017-09-28

**Authors:** Qin Zhao, Feng Guo, Xingshui Zu, Yuchao Chang, Baoqing Li, Xiaobing Yuan

**Affiliations:** 1 Science and Technology on Microsystem Laboratory, Shanghai Institute of Microsystem and Information Technology, Chinese Academy of Sciences, Shanghai 201800, China; qinzhao21@mail.sim.ac.cn (Q.Z.); gfeng7@mail.ustc.edu.cn (F.G.); zuxs@mail.sim.ac.cn (X.Z.); yuchaoch@mail.sim.ac.cn (Y.C.); sinowsn@mail.sim.ac.cn (X.Y.); 2 University of Chinese Academy of Sciences, Beijing 100049, China

**Keywords:** signal enhancement, independent vector analysis, acoustic target classification, microphone array

## Abstract

In this paper, we study how to improve the performance of moving target classification by using an acoustic signal enhancement method based on independent vector analysis (IVA) in the unattended ground sensor (UGS) system. Inspired by the IVA algorithm, we propose an improved IVA method based on a microphone array for acoustic signal enhancement in the wild, which adopts a particular multivariate generalized Gaussian distribution as the source prior, an adaptive variable step strategy for the learning algorithm and discrete cosine transform (DCT) to convert the time domain observed signals to the frequency domain. We term the proposed method as DCT-G-IVA. Moreover, we design a target classification system using the improved IVA method for signal enhancement in the UGS system. Different experiments are conducted to evaluate the proposed method for acoustic signal enhancement by comparing with the baseline methods in our classification system under different wild environments. The experimental results validate the superiority of the DCT-G-IVA enhancement method in the classification system for moving targets in the presence of dynamic wind noise.

## 1. Introduction

In wild environments, the unattended ground sensor (UGS) system is usually employed to acquire military intelligence about intruding targets by detecting and processing their image, acoustic and seismic signals, etc. [1,2]. Compared with other signals, the applications based on acoustic signals provide a simple, portable and easily implementable scheme. Hence, the acoustic signals are widely used in the UGS [3,4,5]. Especially, the classification of moving targets by using of microphone array is of great importance in UGS. However, the acoustic target classification module of the UGS system applied in a wild environment faces a great challenge of complicated and dynamic noise interferences [6].

Mel-frequency cepstral coefficients (MFCCs) are the most widely-used features in speaker and speech recognition [7,8,9]. Meanwhile, we have studied that it can also achieve a satisfactory accuracy in moving acoustic target classification [10,11]. Nevertheless, in the wild environments, the acoustic signals of moving targets are usually contaminated by strong wind noise. Besides, wind noise is ubiquitous since it cannot be totally insulated by windshields. Moreover, the MFCC features are extremely sensitive to the noise interference, which can largely exacerbate the performance of the classification system because of the mismatches between training datasets and test datasets.

To improve the robustness of features and thereupon achieve a satisfactory classification accuracy, the signal enhancement methods are adopted to effectively improve the quality of the acoustic signal by emphasizing the desired component and restraining the interference noise [12,13,14]. The enhancement methods can be categorized into two groups: single-channel techniques and multi-channel techniques. The most-used method with a single microphone is the Wiener filter [15,16], whereas, the amount of noise reduction is in general proportional to the amount of target signal degradation. In addition, the Wiener filter previously required the power spectrum of the signal and noise, which is extremely difficult to achieve in practical application.

Therefore, the enhancement methods based on a microphone array using multi-channel signals are more applicable. The most-used classical methods are the delay-and-sum beamformer (DS) and the minimum variance distortionless response beamformer (MVDR). DS is a widely-used beamforming technology for its simplicity, and several microphone array systems based on DS have been implemented since the 1980s [17,18,19]. Although DS has a simple structure, it requires a large number of microphones to achieve a high performance. However, the microphone arrays in UGS usually consider a relatively small number of microphones. MVDR is very popular for speech enhancement applications [20,21,22], while it is extremely sensitive to the location of source and microphone gains. Thus, it is not suited to moving acoustic targets. Furthermore, to employ DS and MVDR in the microphone array system, the directions of arrival (DOAs) of the acoustic sources are required, then each source signal is separately obtained using the directivity of the array. This means that the beamformers are quite dependent on the estimation of the DOA. Hence, to avoid the limitation of DOA estimation and other disadvantages, blind source separation (BSS) methods have become attractive in the signal enhancement process in recent years [23,24,25,26].

Independent component analysis (ICA) is a classical and the most-used algorithm for the BSS issue [27,28,29], which performs very well in the instantaneous mixing problem, but such a mixing condition is very rare and unrealistic. Actually observed signals are convolutive mixtures in the wild, which means that they are mixed with time delays and convolutions. As an expansion algorithm of the conventional frequency domain ICA [30,31], IVA is designed to retain the dependency within individual source signals, while removing the dependency between different source vectors [32,33]. Instead of a univariate source prior, the dependency in each source vector is retained by adopting a multivariate source prior in IVA. Moreover, IVA separates source signals by estimating an instantaneous unmixing matrix on each frequency bin. It settles the frequency domain BSS problem [34] effectively without suffering from the inherent permutation problem [35] between the frequencies by utilizing the dependencies of frequency bins. From these advantages, IVA is a great method to settle the convolutive blind source separation (CBSS) issues. In the wild environments, the target signal is contaminated by complicated and strong acoustic noise, especially wind noise, which is dynamic and non-additive. When enhancing the moving vehicle signals collected by the microphone array in the wild, it can be considered that observed signals are convolutive mixtures of interference noise and target signal. Hence, we adopt the IVA method to get the enhanced signals for the subsequent moving target classification in UGS.

Furthermore, the nonlinear score function is of vital importance in the IVA algorithm, which can be derived from the source prior [36]. Aside from the multivariate Laplace distribution in the original IVA, several different multivariate distributions have been adopted as the source prior recently [37,38,39]. Especially, the performance of the multivariate generalized Gaussian distribution when it is applied to the IVA application has been studied [40]. In this paper, we adopt a particular multivariate generalized Gaussian distribution as the signal source prior, which can better preserve the dependency across different frequency bins. Moreover, the corresponding nonlinear score function additionally contains an expression about frequency domain energy correlation between the elements of each source vector. Thus, the score function contains more information for the dependency structure, which can thereby better preserve the inter-frequency dependency to improve separation performance. Therefore, our source prior performs better in UGS for moving targets signals, and the experimental results corroborate its superiority.

Moreover, when we employ the improved IVA method for moving target classification in the UGS system, there is also a problem about the contradiction between convergence speed and computational cost in separation process. Therefore, we propose an adaptive variable step strategy in the learning process of the unmixing matrix, which is able to achieve a fast convergence optimization with less computational cost.

In the conventional IVA algorithm, discrete Fourier transform (DFT) is generally used to get the information of the signals about the frequency domain. Here, we convert the time domain signals into the frequency domain using DCT [41] instead of DFT in our work. The reason for choosing DCT is that it is superior to DFT for the transformation of real signals, such as an acoustic signal. For a real signal, DFT gives a complex spectrum and leaves nearly one half of the data unused. In contrast, DCT generates real spectrums for real signals and, hence, avoids the unnecessary computation of redundant data [42], which is more favorable in the UGS system.

Combining a particular multivariate generalized G aussian distribution source prior, an adaptive variable step strategy and DCT with the IVA algorithm, this paper presents a robust signal enhancement method, termed as DCT-G-IVA, which aims to improve the performance of moving target classification in the wild environments. In the meantime, we design a target classification system of moving targets with the improved IVA enhancement method. The experimental results reveal that the classification system with our enhancement method outperforms those with the baseline enhancement methods, indicating the superiority of our proposed method.

In general, this paper has the following contributions:

1. Introducing the IVA algorithm to acoustic signal enhancement based on a microphone array in the wild environments.
2. Presenting an improved IVA method, DCT-G-IVA, which adopts a special multivariate generalized Gaussian distribution as the source prior, an adaptive variable step strategy for learning algorithm. Besides, we employ DCT instead of DFT to convert the time domain observations to the frequency domain.
3. Designing a moving target classification system with the aforementioned DCT-G-IVA enhancement method in UGS and achieving a satisfactory classification accuracy.

Aside from the present section, the remainder of this paper is organized as follows. In Section 2, we formulate the problem that our signal enhancement method solves. Section 3 describes the proposed IVA algorithm, which achieves signal enhancement based on a microphone array in the wild. The system of the target classification is further illustrated in Section 4. In Section 5, the experiments are conducted to evaluate our acoustic signal enhancement method in the target classification system. Concluding remarks are presented in Section 6.

## 2. Signal Model and Problem Formulation

In this section, we formulate the observed signals obtained by the microphone array system. Furthermore, we present the IVA problem for the multichannel acoustic signals. To begin with, we introduce some indispensable notations used in this paper.

- The superscript ∗ denotes the conjugate of the complex number.
- The superscript *H* denotes the Hermitian transpose of the matrix, and the superscript *T* denotes the transpose of the matrix.
- The italic E[·] denotes the statistical expectation.
- The operator det(·) denotes the matrix determinant.
- Plain characters denote scalar variables; boldfaced lowercase characters denote vector variables; and boldfaced uppercase characters denote matrix variables.

We consider a signal model in which an *M*-element microphone array captures convolved observations in the wild. Suppose that *N* source signals $s_{i}\left(t\right),i=1,\ldots ,N$ are mixed and collected at that *M* sensors. The observed signal $x_{j}$ is formed as:

(1) $$x_{j}\left(t\right)=\sum_{i=1}^{N}\sum_{l=1}^{L}a_{ij}\left(l\right)s_{i}\left(t-l\right)+n\left(t\right),\;\;j=1,\ldots ,M$$

where $a_{ij}\left(l\right)$ represents the impulse response from source *i* to sensor *j*, which has *L* length. Here, we assume that the number of sources *N* is already known, and the number of sensors *M* is no less than *N*. Besides, the presence of additive noise n(t) within the above mixing model significantly complicates the BSS problem. It is reduced by applying preprocessing, such as denoising the observed signals through the regularization approach [43]. In the wild environment, because the dominant interference on the moving target signals is wind noise, which is non-additive and convoluted with the source signals, therefore, we do not pay attention to n(t) and drop it in the following work.

The separation system is typically comprised of a set of finite impulse response (FIR) filters $w_{ij}\left(l\right)$ of length *L* to produce *N* separated signals:

(2) $$y_{i}\left(t\right)=\sum_{j=1}^{M}\sum_{l=1}^{L}w_{ij}\left(l\right)x_{j}\left(t-l\right),\;\;i=1,\ldots ,N$$

at the outputs. The separation filters are estimated, and $w_{ij}\left(l\right)$ should be obtained blindly, i.e., without the knowledge of $s_{i}\left(t\right)$ and $a_{ij}\left(t\right)$.

When an observed signal is converted to the frequency domain, the convolution becomes multiplicative in the frequency domain. In the following, *f* denotes the frequency bin, and *F* denotes the number of frequency bins. Moreover, $S^{f}={\left[{s_{1}}^{f},{s_{2}}^{f},\ldots ,{s_{N}}^{f}\right]}^{T}$ means the source signal in the *f*-th frequency bin, and ${s_{n}}^{f}={\left[{s_{n}}^{f}\left(1\right),{s_{n}}^{f}\left(2\right),\ldots ,{s_{n}}^{f}\left(V\right)\right]}^{T}$ is a zero mean source vector; *V* is the number of samples. Meanwhile, to associate components across all channels of the microphone array, the *n*-th source component vector (SCV) is formed by taking each *n*-th component vector from each of the frequency bins, i.e., $S_{n}={\left[{s_{n}}^{1},{s_{n}}^{2},\ldots ,{s_{n}}^{F}\right]}^{T},n=1,2,\ldots ,N$. Here, the different SCVs are independent of each other, and the component vectors of each SCV are related. Then, it can be assumed that the independent sources are linearly mixed into the observations $X^{f}={\left[{x_{1}}^{f},{x_{2}}^{f},\ldots ,{x_{M}}^{f}\right]}^{T}$ in the frequency domain by the M×N unknown non-singular mixing matrix $A^{f}$ in the corresponding frequency bin. Then, we have the matrix representation of the problem as:

(3) $$X^{f}=A^{f}S^{f}\in R^{M\times V},\;\;1\leq f\leq F$$

where $A^{f}$ is the frequency domain response function matrix corresponding to $a_{ij}\left(t\right)$, which should be invertible. The matrix $A=[A^{1},A^{2},\ldots ,A^{F}]$ is called the mixing matrix.

The purpose of the IVA algorithm is to compute the estimation of the source signals. We have the estimated signals $Y^{f}={\left[{y_{1}}^{f},{y_{2}}^{f},\ldots ,{y_{N}}^{f}\right]}^{T}$ in the form as:

(4) $$Y^{f}=W^{f}X^{f}\in R^{N\times V},\;\;1\leq f\leq F$$

where $W^{f}={\left(A^{f}\right)}^{-1}$ is called the unmixing matrix, which separates the mixed signals. The aim of our IVA algorithm is to preferably estimate the unmixing matrix $W=[W^{1},W^{2},\ldots ,W^{F}]$ and then get the separated signal $Y=[Y^{1},Y^{2},\ldots ,Y^{F}]$.

Here, for convenience, we assume that ${x_{i}}^{f}$ is already preprocessed to be zero-mean and white with the eigenvalue decomposition (EVD) from here on, then the rest is to rotate the whiten data to find the independent components.

## 3. The IVA Methods of Signal Enhancement

### 3.1. Independent Vector Analysis

Here, we mainly discuss the cost function and the learning algorithm of IVA. Typically, IVA is achieved by minimizing the mutual information among the estimated SCV as:

(5) $$\begin{array}{cc} J_{IVA}\overset{\Delta }{\;=}I\left[y_{1},\ldots ,y_{N}\right] & =\sum_{i=1}^{N}H\left[y_{n}\right]-H\left[y_{1},\ldots ,y_{N}\right]\\ & =\sum_{i=1}^{N}H\left[y_{n}\right]-H\left[W^{1}X^{1},\ldots ,W^{F}X^{F}\right]\\ & =\sum_{i=1}^{N}H\left[y_{n}\right]-\sum_{f=1}^{F}\log\left|\det\left(W^{f}\right)\right|-C\\ & =\sum_{i=1}^{N}\left(\sum_{f=1}^{F}H\left[{y_{n}}^{f}\right]-I\left(y_{n}\right)\right)-\sum_{f=1}^{F}\log\left|\det\left(W^{f}\right)\right|-C \end{array}$$

where H(·) is the (Shannon relative) entropy [44], which is defined as Equation (6). $I(y_{n})$ is the mutual information within the *n*-th estimated SCV, and *C* is a constant term, which does not depend on the estimated signals, but only on the observations. For the ease of notation, we drop the subscript of $J_{IVA}$ in the rest of this article. Equation (5) shows that minimizing the cost function simultaneously minimizes the entropy of all components and maximizes the mutual information within each SCV. It is also evident that the mutual information is responsible for resolving the permutation ambiguity across multiple datasets, since without the mutual information of the SCVs, the cost function would be identical to that of ICA.

(6) $$H\left[y_{n}\right]=E\left[\log\frac{1}{q\left(y_{n}\right)}\right]=-E\left[\log q\left(y_{n}\right)\right]$$

where $q\left(y_{n}\right)$ is the probability density function (PDF) of vector $y_{n}$. Then, we can rewrite the cost function as:

(7) $$J=-\sum_{f=1}^{F}\log\left|\det\left(W^{f}\right)\right|-\sum_{i=1}^{N}E\left[\log q\left(y_{i}\right)\right]-C$$

When the cost function is minimized, the dependency between different source vectors $s_{i}$ should be removed, but the interrelationship between the components within each vector should be retained. The inter-frequency dependency is modeled by the PDF of the source.

In order to enhance the observed acoustic signals of moving targets, the unmixing matrices are updated in every frequency bin. Let η denote the iteration step in the learning algorithm. The k+1-th update procedure is formulated as:

(8) $$W^{f}\left(k+1\right)=W^{f}\left(k\right)+\eta \Delta W^{f}\left(k\right)$$

To compute the unmixing matrix, we apply the natural gradient (NG), which is well known as a fast convergence method [45]. The natural gradient learning rule is given as:

(9) $$\Delta {w_{ij}}^{f}\left(k\right)=\sum_{n=1}^{N}\left(\Lambda_{in}-{R_{in}}^{f}\right){w_{ij}}^{f}\left(k\right)$$

(10) $${R_{in}}^{f}=E\left[\varphi \left({y_{i}}^{1},{y_{i}}^{2},\ldots ,{y_{i}}^{F}\right)\right]{\left({y_{n}}^{f}\right)}^{*}$$

where $\Delta W^{f}\equiv \left\{\Delta {w_{ij}}^{f}\right\}$ means the gradient matrix. ${R_{in}}^{f}$ is the scored correlation at the current frequency bin; $\Lambda_{ii}$ is equal to ${R_{ii}}^{f}$; and $\Lambda_{in}$ is zero when *i* is not equal to *n*. Moreover, $\varphi^{f}\left({y_{i}}^{1},{y_{i}}^{2},\ldots ,{y_{i}}^{F}\right)$ denotes the nonlinear score function about the *i*-th source in the *f*-th frequency bin, which can be obtained as:

(11) $$\varphi^{f}\left({y_{i}}^{1},{y_{i}}^{2},\ldots ,{y_{i}}^{F}\right)=-\frac{\partial \log\left(q\left({y_{i}}^{1},{y_{i}}^{2},\ldots ,{y_{i}}^{F}\right)\right)}{\partial {y_{i}}^{f}}$$

where $\varphi^{f}$ is the derivative of the logarithm of the source prior.

### 3.2. The Multivariate Generalized Gaussian Source Prior

In order to keep the inter-frequency dependency of each source, the original IVA algorithm exploits a multivariate Laplace distribution as:

(12) $$q\left(s_{i}\right)\propto \exp\left(-\frac{\left|s_{i}-\mu_{i}\right|}{\alpha }\right)$$

where $\mu_{i}$ denotes the mean vector of the *i*-th signal, which is assumed as a zero vector, and α is the scale parameter. In this paper, we consider the IVA with the above multivariate Laplace distribution source prior as the L-IVA method. As such, the nonlinear score function to extract the *i*-th source at the *f*-th frequency bin can be formulated as:

(13) $$\begin{array}{cc} \varphi^{f}\left({y_{i}}^{1},{y_{i}}^{2},\ldots ,{y_{i}}^{F}\right) & =-\frac{\partial \log\left(q\left({y_{i}}^{1},{y_{i}}^{2},\ldots ,{y_{i}}^{F}\right)\right)}{\partial {y_{i}}^{f}}\\ & =\frac{{y_{i}}^{f}}{{\left(\sigma_{i}^{f}\right)}^{2}\sqrt{\sum_{k=1}^{F}\left|\frac{{y_{i}}^{k}}{\sigma_{i}^{k}}\right|}} \end{array}$$

where $\sigma_{i}^{f}$ denotes the standard deviation of the *i*-th source at the *f*-th frequency bin, which is usually set as one. Obviously, Equation (13) is a multivariate function, and the dependency between the frequency bins is thereby accounted for in the learning process.

In our work, we give the formulation of the family of multivariate generalized Gaussian distributions as:

(14) $$q\left(s_{i}\right)\propto \exp\left(-{\left(\frac{{\left(s_{i}-\mu_{i}\right)}^{H}{\sum_{i}}^{-1}\left(s_{i}-\mu_{i}\right)}{\alpha }\right)}^{\beta }\right)$$

where $\alpha ,\beta \in R^{+}$ are separately scale and shape parameters and $\sum_{i}$ is a diagonal matrix, which implies that each frequency bin sample is uncorrelated with the others. If α is set properly, it becomes the multivariate Gaussian distribution when β=1. Moreover, let α=1 and $\beta =\frac{1}{2}$; we can obtain the multivariate Laplace distribution as Equation (12). The previous work in [40] indicates that if β<1, the multivariate generalized Gaussian distribution has a heavier tail, which can have an advantage in separating the nonstationary acoustic signals. Consequently, to apply a better nonlinear score function using Equation (14), β is chosen as $\frac{1}{3}$, and α is chosen as one for simplicity in our IVA method. In the meantime, there is a theoretical demonstration of why a shape parameter of $\frac{1}{3}$ can perform better, and experimental studies found that the best separation performance can be achieved [46].

Moreover, the acoustic signal is real-valued. Then, we have the source prior as:

(15) $$q\left(s_{i}\right)\propto \left(-\sqrt[{3}]{{\left(s_{i}-\mu_{i}\right)}^{T}\sum_{i}^{-1}\left(s_{i}-\mu_{i}\right)}\right)$$

This particular source prior can better preserve the inter-frequency dependencies compared with the original multivariate Laplace source prior. We term the IVA method using the prior of Equation (15) as G-IVA. Then, the nonlinear score function can be derived from the aforementioned source prior as:

(16) $$\varphi^{f}\left({y_{i}}^{1},{y_{i}}^{2},\ldots ,{y_{i}}^{F}\right)=\frac{2}{3}\frac{{y_{i}}^{f}}{{\left(\sigma_{i}^{f}\right)}^{2}\sqrt[{3}]{{\left(\sum_{k=1}^{F}{\left|\frac{{y_{i}}^{k}}{\sigma_{i}^{k}}\right|}^{2}\right)}^{2}}}$$

All the L-IVA and G-IVA methods in this paper adopt an adaptive variable step strategy presented in the following.

### 3.3. Adaptive Variable Step for IVA

Due to slow and poor convergence through nonlinear optimization, the conventional IVA method inherently has a significant disadvantage, particularly when adopting an improper initialization in the learning process of unmixing matrix. To be specific, when a large step size is set in the learning algorithm, a fast convergence speed may be acquired, while the optimal solution is also easy to miss. However, with a small step size, the global optimal solution can be reached; meanwhile, a slow convergence speed will arise. Even worse, the learning process may be too slow to reach convergence, which leads to the failure of the separation.

In order to make a good tradeoff between the computational cost and fast-convergence optimization, here we propose an adaptive variable step strategy in the updating process of the unmixing matrix $W^{f}$, instead of the fixed iterative step size in the conventional IVA. The adaptive step size adjustment method is put forward as:

(17) $$\eta \left(k\right)=c_{1}\left|1-e^{c_{2}\cdot \Delta J}\right|$$

where ΔJ=J(k+1)−J(k) is the control variable and $c_{1}$ and $c_{2}$ are two empirical parameters. With the above adaptive variable step strategy, our IVA algorithm can automatically select the step size to achieve a faster convergence with a lesser iteration number. In addition, this adaptive variable step strategy is easy to implement with a low computational cost, which is suited to the UGS system.

#### Parameters’ Selection

We have proposed an adaptive variable step strategy for the IVA learning process. Here, we discuss how to select the empirical parameters $c_{1}$ and $c_{2}$. We choose two speech signals from the TIMIT database [47] as the source signals. After implementing a randomly convolutive mixing, we conduct the adaptive IVA method with the adaptive variable step strategy to separate the mixtures. The separation performance is assessed using the inter-symbol-interference (ISI), which can be formulated as:

(18) $$\mathrm{ISI}\left(R\right)=\frac{1}{N}\sum_{i=1}^{N}\left(\sum_{j=1}^{N}\frac{\left|R_{ij}\right|}{max_{k}\left|R_{ik}\right|}-1\right)+\frac{1}{N}\sum_{j=1}^{N}\left(\sum_{i=1}^{N}\frac{\left|R_{ij}\right|}{max_{k}\left|R_{kj}\right|}-1\right)$$

where *R* is the correlation matrix of the original speech signals and the separated signals and $R_{ik}$ means the correlation coefficient of the *i*-th source and the *k*-th separated signal. Notation ${max}_{k}\left|R_{ik}\right|$ denotes the absolute value of the largest element in each row of *R*, which corresponds to the source signal.

Ideally, the correlation coefficient of a source and the corresponding separated signal equals one, and the $\mathrm{ISI}\left(R\right)$ equals zero. In our simulation experiments, the parameter $c_{1}$ is fixed at 1.15, and $c_{2}$ is varied from 0.01–2; the results are shown in Table 1. Moreover, we also make a comparison of iterations for convergence. During the learning process, The IVA algorithm runs until the decrement of the cost function ΔJ is less than $10^{-9}$. When $c_{2}$ is greater than three, the adaptive IVA method fails to achieve convergence.

From Table 1, we can notice that the appropriate range of values for $c_{2}$ is 1.2–1.4. Moreover, we fix the parameter $c_{2}$ to 1.22 and vary $c_{1}$ from 0.1–3. The results are shown in Table 2, from which we find that the appropriate range of values for $c_{1}$ to be 1.0–1.2. Besides, when $c_{1}$ is greater than four, the adaptive IVA method fails to achieve convergence. The adaptive IVA algorithm is not very sensitive to $c_{1}$ and $c_{2}$, yet proper parameters are required to be selected. For comparison, the IVA method with a fixed iterative step needs more than 100 iterations to reach convergence.

### 3.4. DCT versus DFT

From a computational viewpoint, differing from the DFT transform using sine and cosine functions, DCT uses only cosine functions to express the signal. Typically, the DCT X(k) of the time domain signal x(n) is formulated as:

(19) $$X\left(k\right)=c_{d}\left(k\right)\sum_{n=1}^{N}x\left(n\right)\cos\left[\frac{\pi (2n-1)(k-1)}{2N}\right]$$

where *N* denotes the length of the time domain signal. Furthermore, the inverse discrete cosine transform (IDCT) is easier. By summarizing the results of the base function in the frequency domain multiplying the corresponding amplitude, we can get the corresponding values of the time domain elements. The IDCT x(n) of the frequency domain signal X(k) is computed by:

(20) $$x\left(n\right)=\sum_{k=1}^{N}c_{d}\left(k\right)X\left(k\right)\cos\left[\frac{\pi (2n-1)(k-1)}{2N}\right]$$

where *k* is the frequency bin index, and the coefficient $c_{d}\left(k\right)$ is given by:

(21) $$c_{d}\left(k\right)=\left\{\begin{array}{cc} \frac{1}{\sqrt{N}} & \mathrm{if}\;k=1\\ \sqrt{\frac{2}{N}} & \mathrm{if}\;2\leq k\leq N \end{array}\right.$$

The energy of most natural signals, such as acoustic signals and image signals, are concentrated in the low frequency domain. The decorrelation performance of the DCT is close to the Karhunen–Loeve (KL) transform, which has the optimal property of decorrelation [48]. Moreover, DCT coefficients have a better “energy concentration” feature than DFT coefficients, namely we can express the time domain data x(n) with a lesser number of DCT coefficients.

From a practical viewpoint, although the computational complexities of DFT and DCT both are $O\left(N^{2}\right)$, DCT only uses cosine functions to express the signal, namely it consumes about half the computation and memory that DFT consumes. Furthermore, IDCT has the ability to reconstruct the time domain data of acoustic signals better. Considering such advantages of DCT, we adopt the short-time DCT to get the frequency domain information of the acoustic signals for our application.

## 4. The System of Moving Target Classification

**Figure 1:**
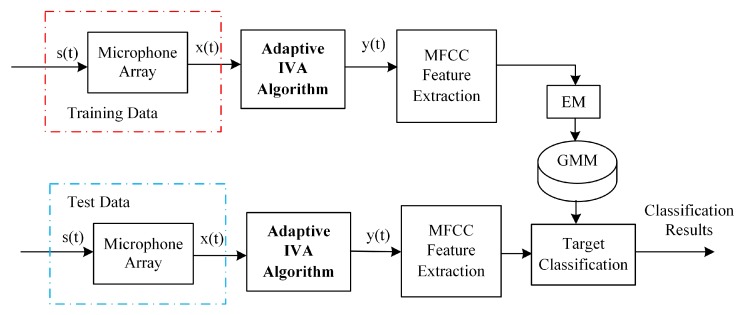
Diagram of the target classification system with the independent vector analysis (IVA) algorithm.

In this section, we design a classification system for moving targets based on the microphone array and proposed improved IVA method in the wild. The purpose of our study is to provide a reliable and stable classification system of moving targets using the proposed DCT-G-IVA method. This system focuses on the DCT-G-IVA method, which enhances the acoustic signals in the presence of dynamic wind noise in the UGS system. The implemented system of the moving target classification is demonstrated in Figure 1. We evaluate the proposed enhancement algorithm for acoustic signal enhancement by comparing with the baseline enhancement methods in the classification framework of MFCC + GMM, which is composed of the classic feature MFCC and the popular classifier Gaussian mixture model (GMM) [49,50].

While using the IVA algorithm to enhance the observed signal, we consider the actual observed signal as a convolutive mixture of the real target signal and other interference signals. The system presented in Figure 1 starts with the adaptive IVA algorithm for enhancing the observations of the moving targets obtained by the microphone array. Because the moving vehicle signals have been well enhanced, the MFCC features are more robust, and the classification performance can be improved effectively. There is a significant advantage of using IVA that it does not require much a priori knowledge of the target signal and the noise signal. Hence, the IVA method is suited for the acoustic signal enhancement in the wild, where the interference noise is dynamic and complicated.

**Figure 2:**
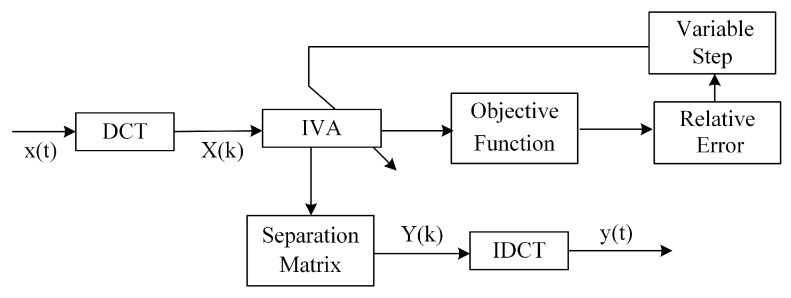
Block diagram of the DCT-G-IVA method.

In the IVA methods, the slow convergence can generate signal distortion, then result in a separation failure. Thus, we propose the adaptive variable step approach during updating the unmixing matrix in our IVA method, which is demonstrated in Figure 2. It is noteworthy that we also use a particular multivariate generalized Gaussian distribution as the source prior as the G-IVA. To validate their performance, we have studied the comparison between G-IVA and the original L-IVA in our classification experiments specified in Section 5. Meanwhile, we adopt DCT instead of DFT in the original IVA algorithm to reduce the computation and memory cost in UGS.

## 5. Experiments and Results

### 5.1. Experimental Description

**Figure 3:**
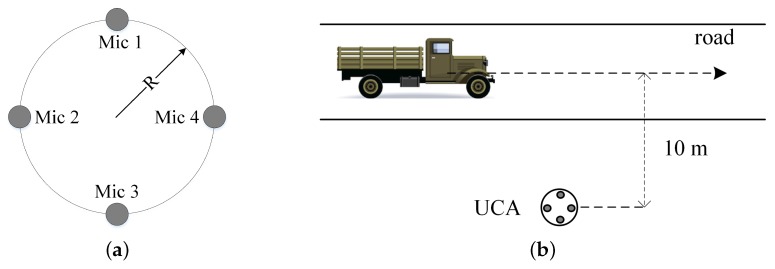
Illustration of the experimental scenario. (**a**) The arrangement of the four-element microphone uniform circular array (UCA); (**b**) Layout of the experiment in the wild.

**Figure 4:**
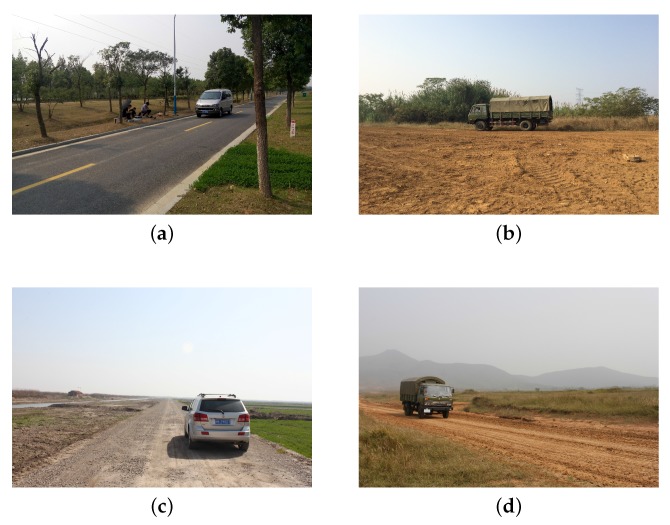
Four different experimental environments in the wild. (**a**) Cement road; (**b**) Dirt road; (**c**) Gravel road; (**d**) Mud road.

In this paper, we collected our acoustic samples of observed signals by the microphone array developed in [51,52,53]. Combining the microelectromechanical systems (MEMS) technology with the uniform circular array (UCA), a four-element MEMS microphone (ADMP504) UCA with a radius of R=0.02 m was deployed. Then, the inputs of these microphones were transferred into separate channels of a four-channel 16-bit simultaneous ADC, which was sampled at a rate of 8192 Hz. The microphone array system we adopted is shown in Figure 3a, and the experimental scenario is also depicted in Figure 3b, where the UCA was placed 10 m away from the road. In the meantime, the different experimental environments are shown in Figure 4.

In our IVA-based separation process for signal enhancement, we use a 1024-point DCT and Hanning window to convert the time domain observed signals to the frequency domain. The length of the window is 1024 with a 75% overlap. Besides, a 1024-point fast Fourier transform (FFT) is used to compute DFT in the STFT domain to do a comparison. When the adaptive variable step strategy is using, the two empirical parameters $c_{1}$ and $c_{2}$ are separately set as 1.15 and 1.22 in the subsequent experimental confirmation. During the learning process, the IVA algorithms run until the decrement of the cost function ΔJ is less than $10^{-9}$. Besides, we make the comparison among DCT-G-IVA, DFT-G-IVA, DCT-L-IVA and DFT-L-IVA in our classification experiments.

In the classification system, the number of filters in MFCC is 24 in the feature extraction. While the model is training, the number of the Gaussian functions in GMM is 10. Such a combination could achieve an optimal performance in the experiments.

### 5.2. Datasets

To show the robustness of our improved IVA algorithms in the signal enhancement application, experiments are conducted using a total of hundreds of sample signals collected under different wild environments. The composition of our labeled sample set is shown in Table 3.

In our experiment, we have collected no less than 224 audio signals from three kinds of vehicles and noise. Therein, tracked vehicles (TV) usually have the longest detection distance. The time duration of each collected audio sample is different; thus, we consider a frame-based classification. Thus, in the classification process, each audio sample is divided into frames, and the frame size is 1024 with no overlap between adjacent frames.

**Figure 5:**
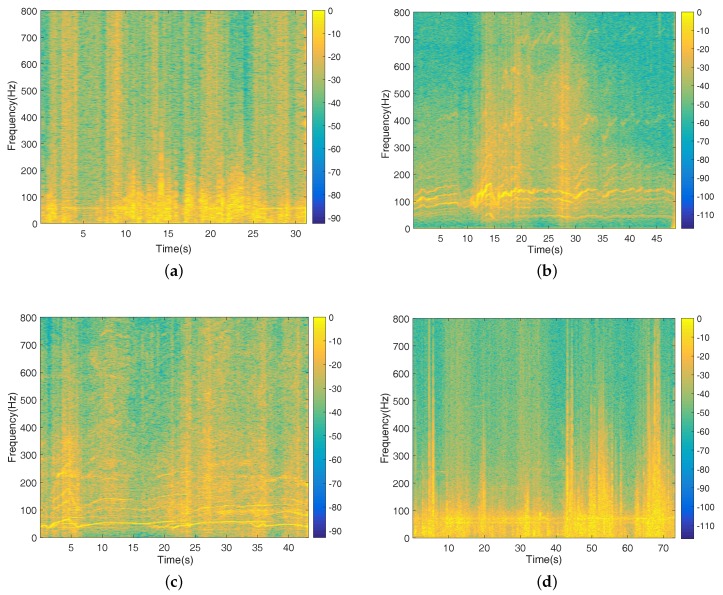
Spectrums of acoustic signals collected in Nanjing. (**a**) Spectrum of a noise signal; (**b**) Spectrum of a tracked vehicle (TV) signal; (**c**) Spectrum of a truck signal; (**d**) Spectrum of a car signal.

Figure 5 demonstrates the spectrums of the noise signal and different vehicle signals, which were collected in a suburban district around Nanjing in December 2015, and the wind power level was around five. From Figure 5a, we notice that the spectrum of wild noise (mostly wind noise) covers a wide range. Actually, interference noise is ubiquitous in real-world environments and can seriously impair the quality and intelligibility of target signals. Meanwhile, it is noticeable that the spectrum of the moving vehicle target mainly resides in the low frequency part in the Figure 5b–d. Moreover, The spectral content of a vehicle signal is approximately regular and is mainly dominated by the engine and exhaust system [54]. The spectral contents of interest are composed of a limited number of pitches and harmonics; thus, we can notice that there are some distinct spectrum lines in the spectrums of vehicle signals. An unknown target can hardly be recognized if its harmonics are contaminated by wind noise. In Figure 5, we can almost distinguish the spectrum lines of TV and truck in the presence of wind noise, while the spectrum lines of car are difficult to find, which indicates that the noise contaminates the vehicle signals in the wild environments.

### 5.3. Result Analysis

In our classification experiments, we train the classifiers on the training sets with different sizes to get a comprehensive comparison [55]. Namely, we randomly select the training sets from our labeled datasets at different percentages. Moreover, ten-fold cross-validation is employed to evaluate the classification performance. The classification accuracies of our classification experiments are presented in Table 4. As mentioned previously, the results of target classification are frame-based.

Classification experiment results in Table 4 have validated the superiority of adopting the proposed DCT-G-IVA method for signal enhancement. For comparison, we also adopt two microphone array-based enhancement methods of DS and the average-channel signal as baseline algorithms. DS is a classical approach for signal enhancement with microphone array sensors, and we employ the multiple signal classification (MUSIC) algorithm to estimate the DOAs for DS [56]. Besides, the average-channel signal is obtained by $s=\frac{1}{N}\sum_{i=1}^{M}s_{i}$, where $s_{i}$ denotes the acoustic signal of the *i*-th channel with an *M*-element microphone array. With the results exhibited in Table 4, compared with the baseline enhancement algorithms, our IVA-based methods contribute better to the classification. Moreover, DCT is preferable to DFT in the transformation of the acoustic signal. Hence, the results of the last four columns in Table 4 present that the IVA methods adopting DCT perform better in the classification experiments. Particularly, the classification accuracy with the DCT-G-IVA method achieves an excellent result, which is up to 96.33%.

**Figure 6:**
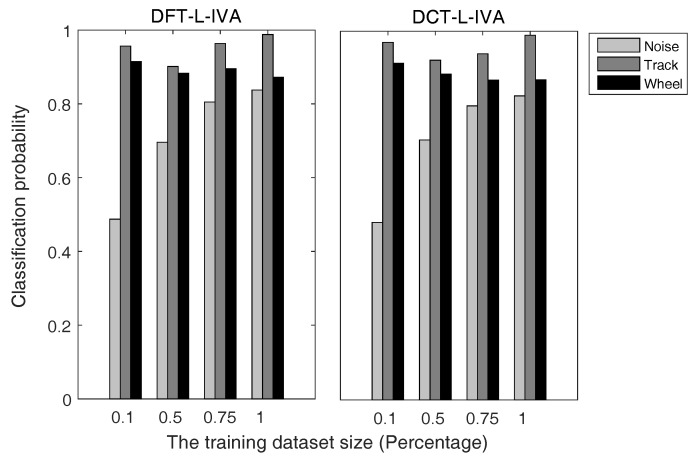
Classification probabilities of each target type using L-IVA on the training sets with different sizes.

**Figure 7:**
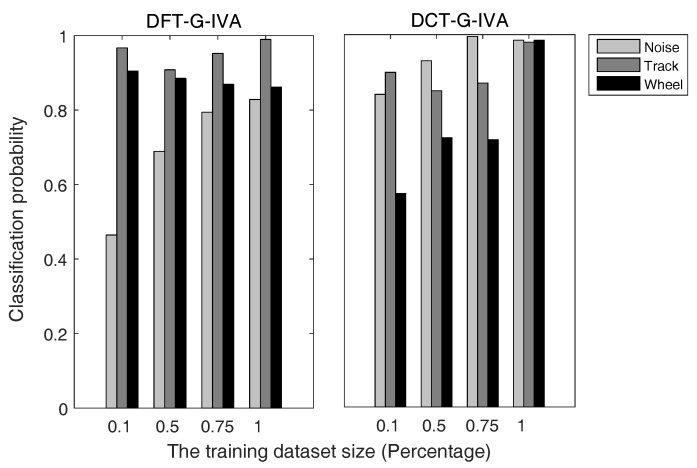
Classification probabilities of each target type using G-IVA on the training sets with different sizes.

Figure 6 is the comparison of the classification probabilities of each target type using two L-IVA signal enhancement methods, which use the DFT and DCT to get the frequency domain information of moving target signals. Likewise, Figure 7 shows the comparison of the classification probabilities of each target type using two G-IVA signal enhancement methods, which use the DFT and DCT to get the frequency domain information of moving target signals. The horizontal axis and vertical axis of Figure 6 and Figure 7 represent the percentages of the training sets in our datasets and the classification probabilities of noise, TV and wheeled vehicles. Here, the wheeled vehicles refer to the car and truck in Table 3. Here, we randomly select the training sets from our labeled datasets at different percentages, while the test sets are the remaining sample data. The classification probability of each type denotes the mean probability of correct classification for all target signals of this type, which can reflect the classification performance from another perspective. Besides, the results of classification probabilities are signal-based.

Because the observed signal is a mixture of many signals and is a more Gaussian signal due to the central limit theorem, the multivariate generalized Gaussian distribution source prior can model the signals more accurately and exploit the frequency domain energy correlation within each source vector. Moreover, the particular multivariate generalized Gaussian distribution we adopted has a heavier tail compared with the original multivariate Laplace distribution, and it can preserve the dependency through different frequency bins to utilize more information describing the dependency structure and provide improved source separation performance. This accounts for the results in Figure 6 and Figure 7 and Table 4 that the G-IVA algorithms perform better than the L-IVA algorithms. Furthermore, DCT-G-IVA presents the best performance in the Figure 7, which achieves a wonderful result such that the mean classification probabilities of noise, tracked vehicle and wheeled vehicle are respectively up to 0.9850, 0.9794, and 0.9845.

All of the experiments were executed in the MATLAB R2015b environment of an industrial computer (8-core, 3.6-GHz frequency and 8-GB memory) to process the datasets detailed in Section 5.2. The execution times of separation using the four IVA methods are shown in Table 5. Furthermore, the convergence of the adaptive variable step IVA was very fast, taking less than 38 iterations. To be specific, the G-IVA only took 34 iterations, while the L-IVA took 37 iterations for convergence. In contrast, the IVA with a fixed step took around 300 iterations to converge. Besides, DCT only uses cosine functions to express the signal, namely it consumes about half the computation and memory of the DFT. Hence, our proposed DCT-G-IVA method can achieve a better enhancement performance, thereupon getting a higher classification accuracy with a lower computational consumption. The results in Table 4 and Table 5 validate the above points.

In addition, when using FFT to compute DFT coefficients, the sample size needs to be limited to $2^{n},n\in Z^{+}$, even though it can be solved by using zero padding. However, DCT does not limit the sample size, which is more convenient and applicable for the hardware implementation in the UGS system.

## 6. Conclusions

In this paper, a method for acoustic signal enhancement in the wild based on a microphone array and IVA has been proposed. We term our proposed method DCT-G-IVA, which adopts a multivariate generalized Gaussian distribution as the source prior, an adaptive variable step strategy for the learning algorithm and DCT instead of DFT to convert the time domain observed signals to the frequency domain. Moreover, we employ the improved IVA method on the UGS system to enhance the acoustic signals collected by the microphone array, thereupon classify the moving targets using the enhanced signals. Experiments are conducted to evaluate the DCT-G-IVA method for acoustic signal enhancement by comparing with the baseline methods in the classification system of MFCC+GMM under different wild environments. According to the experimental results, the adaptive G-IVA and L-IVA algorithms outperform the classic baseline enhancement methods in the wild environment with wind noise existing. Especially DCT-G-IVA performs best with the highest classification accuracy of 96.33% and relatively less computational cost. Finally, the results suggest that the proposed signal enhancement method is very suited to the microphone array deployed in UGS with a high classification accuracy and a good reliability to resist dynamic wind noise and other interferences. Furthermore, the proposed method could also provide an inspiration to other applications such as speaker recognition in a noisy environment.

## Figures and Tables

**Table 1:** The separation performance with varying $c_{2}$.

c2	0.01	0.2	0.4	0.6	0.8	1.0	1.2	1.4	1.6	1.8	2.0
**Iterations**	17	37	37	35	34	31	25	24	24	24	24
**ISI**	0.129	0.125	0.124	0.121	0.117	0.112	0.091	0.103	0.105	0.114	0.126

**Table 2:** The separation performance with varying $c_{1}$.

c1	0.1	0.3	0.5	0.8	1.0	1.2	1.4	1.8	2.0	2.5	3.0
**Iterations**	29	31	33	31	27	26	22	21	19	18	18
**ISI**	0.125	0.123	0.115	0.100	0.094	0.095	0.112	0.117	0.122	0.178	0.243

**Table 3:** The composition of our labeled sample set.

Target	Car	Truck	TV	Noise	Sum
**Sample number**	58	46	49	71	**224**
**Frame number**	6240	8551	12,185	11,237	**38,213**

**Table 4:** Classification accuracies of moving targets in the wild. DS, delay-and-sum beamformer.

Training Set	Average	DS	DFT-L-IVA	DCT-L-IVA	DFT-G-IVA	DCT-G-IVA
0.1	0.7937	0.8083	0.8901	0.8964	0.8912	**0.8987**
0.5	0.8476	0.8457	0.9373	0.9419	0.9482	**0.9536**
0.75	0.8572	0.8577	0.9354	0.9356	0.9421	**0.9570**
1	0.8636	0.8656	0.9482	0.9507	0.9589	**0.9633**

**Table 5:** The execution time of separation.

Methods	DFT-L-IVA	DCT-L-IVA	DFT-G-IVA	DCT-G-IVA
**Execution time**	344.013 s	295.059 s	325.680 s	198.953 s

## References

[1] Winston M., Mcquiddy J., Jones B. Affordable Next-generation UGS Development and Testing. Proceedings of the SPIE—The International Society for Optical Engineering.

[2] Chattopadhyay P., Ray A., Damarla T. (2016). Simultaneous tracking and counting of targets in a sensor network. J. Acoust. Soc. Am..

[3] William P.E., Hoffman M.W. (2011). Classification of Military Ground Vehicles Using Time Domain Harmonics’ Amplitudes. IEEE Trans. Instrum. Meas..

[4] Zu X., Guo F., Huang J., Zhao Q., Liu H., Li B., Yuan X. (2017). Design of an Acoustic Target Intrusion Detection System Based on Small-Aperture Microphone Array. Sensors.

[5] Zu X., Zhang S., Guo F., Zhao Q., Zhang X., You X., Liu H., Li B., Yuan X. (2017). Vehicle Counting and Moving Direction Identification Based on Small-Aperture Microphone Array. Sensors.

[6] Guo F., Liu H., Huang J., Zhang X., Zu X., Li B., Yuan X. (2016). Design of a Direction-of-Arrival Estimation Method Used for an Automatic Bearing Tracking System. Sensors.

[7] Sahidullah M., Saha G. (2013). A Novel Windowing Technique for Efficient Computation of MFCC for Speaker Recognition. IEEE Signal Process. Lett..

[8] Nakagawa S., Wang L., Ohtsuka S. (2012). Speaker Identification and Verification by Combining MFCC and Phase Information. IEEE Trans. Audio Speech Lang. Process..

[9] Alsina-Pagés R.M., Navarro J., Alías F., Hervás M. (2017). HomeSound: Real-Time Audio Event Detection Based on High Performance Computing for Behaviour and Surveillance Remote Monitoring. Sensors.

[10] Huang J., Xiao S., Zhou Q., Guo F., You X., Li H., Li B. (2015). A Robust Feature Extraction Algorithm for the Classification of Acoustic Targets in Wild Environments. Circuits Syst. Signal Process..

[11] Guo F., Huang J., Zhang X., You X., Zu X., Zhao Q., Ding Y., Liu H., Li B. (2017). A Classification Method for Moving Targets in the Wild Based on Microphone Array and Linear Sparse Auto-Encoder. Neurocomputing.

[12] Wang J.C., Lee H.P., Wang J.F., Lin C.B. (2008). Robust Environmental Sound Recognition for Home Automation. IEEE Trans. Autom. Sci. Eng..

[13] Choi J.H., Chang J.H. (2012). On using acoustic environment classification for statistical model-based speech enhancement. Speech Commun..

[14] Li Y., Ho K.C., Popescu M. (2012). A microphone array system for automatic fall detection. IEEE Trans. Biomed. Eng..

[15] Chen J., Benesty J., Huang Y., Doclo S. (2006). New insights into the noise reduction Wiener filter. IEEE Trans. Audio Speech Lang. Process..

[16] Yang F., Wu M., Yang J. (2012). Stereophonic Acoustic Echo Suppression Based on Wiener Filter in the Short-Time Fourier Transform Domain. IEEE Signal Process. Lett..

[17] Flanagan J.L., Johnston J.D., Zahn R., Elko G.W. (1985). Computer-steered microphone arrays for sound transduction in large rooms. J. Acoust. Soc. Am..

[18] Benesty J., Chen J., Huang Y. (2008). Conventional Beamforming Techniques.

[19] Zeng Y., Hendriks R.C. (2014). Distributed Delay and Sum Beamformer for Speech Enhancement via Randomized Gossip. IEEE/ACM Trans. Audio Speech Lang. Process..

[20] Habets E.A.P., Benesty J., Cohen I., Gannot S., Dmochowski J. (2010). New Insights Into the MVDR Beamformer in Room Acoustics. IEEE Trans. Audio Speech Lang. Process..

[21] Zhang J., Wang Q., Wang X.Q., Yuan C.M. Simulation of high-speed train pass-by noise source identification based on MVDR beamforming. Proceedings of the IEEE International Conference on Control and Automation.

[22] Pan C., Chen J., Benesty J. (2014). Performance Study of the MVDR Beamformer as a Function of the Source Incidence Angle. IEEE/ACM Trans. Audio Speech Lang. Process..

[23] Haykin S. (2000). Unsupervised Adaptive Filtering Volume I: Blind Source Separation.

[24] Visser E., Otsuka M., Lee T.W. (2003). A spatio-temporal speech enhancement scheme for robust speech recognition in noisy environments. Speech Commun..

[25] Souden M., Araki S., Kinoshita K., Nakatani T., Sawada H. (2013). A Multichannel MMSE-Based Framework for Speech Source Separation and Noise Reduction. IEEE Trans. Audio Speech Lang. Process..

[26] Zoulikha M., Djendi M. (2016). A new regularized forward blind source separation algorithm for automatic speech quality enhancement. Appl. Acoust..

[27] Hyvärinen A. (1999). Fast and robust fixed-point algorithms for independent component analysis. IEEE Trans. Neural Netw..

[28] Comon P., Jutten C. (2010). Handbook of Blind Source Separation: Independent Component Analysis and Separation.

[29] Hyvärinen A. (2012). Independent component analysis: Recent advances. Philos. Trans. R. Soc. A.

[30] Bell A.J., Lambert R.H. Blind Separation of Delayed and Convolved Sources. Proceedings of the Conference on Neural Information Processing Systems (NIPS 1996).

[31] Smaragdis P. (1998). Blind separation of convolved mixtures in the frequency domain. Neurocomputing.

[32] Lee I., Kim T., Lee T.W. (2007). Fast fixed-point independent vector analysis algorithms for convolutive blind source separation. Signal Process..

[33] Anderson M., Fu G.S., Phlypo R., Adali T. (2014). Independent Vector Analysis: Identification Conditions and Performance Bounds. IEEE Trans. Signal Process..

[34] Sawada H., Mukai R., Araki S., Makino S. (2005). Frequency-Domain Blind Source Separation.

[35] Hiroe A. (2006). Solution of Permutation Problem in Frequency Domain ICA, Using Multivariate Probability Density Functions. Lect. Notes Comput. Sci..

[36] Kim T., Attias H.T., Lee S.Y., Lee T.W. (2007). Blind Source Separation Exploiting Higher-Order Frequency Dependencies. IEEE Trans. Audio Speech Lang. Process..

[37] Harris J., Rivet B., Naqvi S.M., Chambers J.A., Jutten C. Real-time independent vector analysis with Student’s t source prior for convolutive speech mixtures. Proceedings of the IEEE International Conference on Acoustics, Speech and Signal Processing.

[38] Anderson M., Adali T., Li X.L. (2012). Joint Blind Source Separation With Multivariate Gaussian Model: Algorithms and Performance Analysis. IEEE Trans. Signal Process..

[39] Giri R., Rao B.D., Garudadri H. (2017). Reweighted Algorithms for Independent Vector Analysis. IEEE Signal Process. Lett..

[40] Boukouvalas Z., Fu G.S., Adali T. An efficient multivariate generalized Gaussian distribution estimator: Application to IVA. Proceedings of the 2015 49th Annual Conference on Information Sciences and Systems (CISS).

[41] Ahmed N.N., Natarajan T., Rao K.R. (1974). Discrete Cosine Transform. IEEE Trans. Comput..

[42] Strang G. (1999). The Discrete Cosine Transform. Siam Rev..

[43] Rhabi M.E., Fenniri H., Keziou A., Moreau E. (2013). A robust algorithm for convolutive blind source separation in presence of noise. Signal Process..

[44] Yang Y. (2012). Elements of Information Theory. J. Am. Stat. Assoc..

[45] Amari S.I., Cichocki A., Yang H.H. A New Learning Algorithm for Blind Signal Separation. Proceedings of the 8th International Conference on Neural Information Processing Systems.

[46] Liang Y., Harris J., Naqvi S.M., Chen G., Chambers J.A. (2014). Independent vector analysis with a generalized multivariate Gaussian source prior for frequency domain blind source separation. Signal Process..

[47] Garofolo J.S., Lamel L.F., Fisher W.M., Fiscus J.G., Pallett D.S., Dahlgren N.L., Zue V. (1993). TIMIT Acoustic-Phonetic Continuous Speech Corpus.

[48] Proakis J.G., Manolakis D.G. (2007). Digital Signal Processing: Principles, Algorithms and Applications.

[49] Grimaldi M., Cummins F. (2008). Speaker Identification Using Instantaneous Frequencies. IEEE Trans. Audio Speech Lang. Process..

[50] Jayanna H.S., Prasanna S.R.M. (2009). An experimental comparison of modelling techniques for speaker recognition under limited data condition. Sadhana.

[51] Zhang X., Huang J., Song E., Liu H., Li B., Yuan X. (2014). Design of Small MEMS Microphone Array Systems for Direction Finding of Outdoors Moving Vehicles. Sensors.

[52] Guo F., Huang J., Zhang X., Cheng Y., Liu H., Li B. (2015). A Two-Stage Detection Method for Moving Targets in the Wild Based on Microphone Array. IEEE Sens. J..

[53] Huang J., Zhang X., Guo F., Zhou Q., Liu H., Li B. (2015). Design of an Acoustic Target Classification System Based on Small-Aperture Microphone Array. IEEE Trans. Instrum. Meas..

[54] Cevher V., Chellappa R., Mcclellan J.H. (2009). Vehicle Speed Estimation Using Acoustic Wave Patterns. IEEE Trans. Signal Process..

[55] Raina R., Battle A., Lee H., Packer B., Ng A.Y. Self-taught learning: Transfer learning from unlabeled data. Proceedings of the International Conference on Machine Learning.

[56] Li J., Zhang X., Cao R., Zhou M. (2013). Reduced-Dimension MUSIC for Angle and Array Gain-Phase Error Estimation in Bistatic MIMO Radar. IEEE Commun. Lett..

